# Psychotherapy training in diverse cultural and institutional contexts: challenges and adaptations

**DOI:** 10.1192/j.eurpsy.2026.10307

**Published:** 2026-07-31

**Authors:** A. N. Alhadi

**Affiliations:** Psychiatry Department, College of Medicine, King Saud University, Riyadh, Saudi Arabia

## Abstract

**Abstract:**

Psychotherapy training is often based on models developed within specific cultural and institutional contexts, which may not readily translate across diverse settings. This presentation explores key challenges in implementing psychotherapy training in varied cultural, social, and healthcare environments, including differences in values, language, resources, and institutional structures. It highlights practical adaptation strategies that balance fidelity to evidence-based psychotherapies with cultural relevance and feasibility. Emphasis is placed on culturally responsive, competency-based training approaches to strengthen the global psychotherapy workforce.

**Disclosure of Interest:**

None Declared

